# Peptide-Enabled Targeted Delivery Systems for Therapeutic Applications

**DOI:** 10.3389/fbioe.2021.701504

**Published:** 2021-07-01

**Authors:** Mingpeng Liu, Xiaocui Fang, Yanlian Yang, Chen Wang

**Affiliations:** ^1^CAS Key Laboratory of Biological Effects of Nanomaterials and Nanosafety, CAS Key Laboratory of Standardization and Measurement for Nanotechnology, CAS Center for Excellence in Nanoscience, National Center for Nanoscience and Technology, Beijing, China; ^2^Department of Chemistry, Tsinghua University, Beijing, China; ^3^University of Chinese Academy of Sciences, Beijing, China

**Keywords:** targeting peptide, nanostructure, enhanced receptor-specificity, drug delivery, tumor therapy

## Abstract

Receptor-targeting peptides have been extensively pursued for improving binding specificity and effective accumulation of drugs at the site of interest, and have remained challenging for extensive research efforts relating to chemotherapy in cancer treatments. By chemically linking a ligand of interest to drug-loaded nanocarriers, active targeting systems could be constructed. Peptide-functionalized nanostructures have been extensively pursued for biomedical applications, including drug delivery, biological imaging, liquid biopsy, and targeted therapies, and widely recognized as candidates of novel therapeutics due to their high specificity, well biocompatibility, and easy availability. We will endeavor to review a variety of strategies that have been demonstrated for improving receptor-specificity of the drug-loaded nanoscale structures using peptide ligands targeting tumor-related receptors. The effort could illustrate that the synergism of nano-sized structures with receptor-targeting peptides could lead to enrichment of biofunctions of nanostructures.

## Introduction

Traditional small molecule drugs often suffer from various biopharmaceutical delivery obstacles such as non-specific distribution and inadequate accumulation at the site of interest. These limitations could be overcome by using appropriate strategies for directing drugs to specific disease tissues. In this field, nanotechnology has illustrated immense potentials in the past few decades, such as controlled drug release, promoted targeting drug delivery, and so on (Patra et al., 2018). Compared with bulk materials, nanostructures possess ultra-small sizes and large specific surface areas that can distinctively interact with biological interfaces. Their unique properties and benefits over bulk materials have been widely explored and exploited in the biomedical fields (McNamara and Tofail, 2017; Han et al., 2019).

Generally, the strategies based on nanostructures for effective drug delivery to the destination parts mainly include passive and active targeting. The passive targeting is mainly achieved by the enhanced permeability and retention (EPR) effect, which could be attributed to the abnormal structures of tumor vessels (Matsumura and Maeda, 1986). However, the therapeutic efficacies of cancer therapies based on passive targeting are far from optimal. One of the reasons is that the EPR effect is yet to be rigorously established and has limitations due to the EPR effect is highly diverse across both different tumor types and different subregions of a single tumor tissue (Bjornmalm et al., 2017).

The practicability of active targeting tumor therapy has been verified in many studies. By introducing receptor-specific ligands such as antibodies, aptamers, small molecules, and peptides, nanostructures with active targeting capability can be constructed. These active targeting delivery systems can direct payloads to the tumor parenchyma by specific recognition between tumor-associated antigens and targeting ligands. Among these receptor-specific ligands, antibodies and other large protein-based ligands possess the best binding affinity, but they usually suffer from several limitations including the immunogenicity, large size (∼ 150 KDa) and non-specific clearance by the reticuloendothelial system (RES) and liver, resulting in poor passive diffusion across capillary endothelial cell membranes and dose-limiting systemic toxicity (Aina et al., 2002). Therefore, the successful application of macromolecular compounds, such as monoclonal antibodies, is limited to the vascular endothelium tumor (Thorpe, 2004) and hematological malignancies (Reff et al., 2002).

In comparison, bioactive peptides have a much smaller molecular weight (<10 KDa). Despite only 1∼10% of binding affinity compared with parent antibodies (Zhang et al., 2012), peptides provide superior advantages including lower immunogenicity, stronger penetration, lower production cost, and easier synthesis and modification. Additionally, the large specific surface area of nanostructures allows peptides to present multiple copies at the same time to obtain higher binding affinity, and also offers the possibility to incorporate different peptide ligands in a single construct. Compared with small molecule ligands, peptides have higher diversity, specificity, and targeting capability (Vlieghe et al., 2010; Sasikumar and Ramachandra, 2018; Ganesan et al., 2019). With these factors, peptides have been widely investigated as candidates for novel drugs.

At the same time, peptide drugs also suffer from unfavorable obstacles for clinical application including susceptibility to digestion by proteases and rapid plasma clearance (several minutes to several hours of half-life). To overcome these obstacles, various nanostructures have been utilized as carriers for peptide drug delivery, which could enhance solubility and circulation lifetime of peptides, and promote their specific accumulation into target tissues (Vo et al., 2012; Maeda et al., 2013; Bozzuto and Molinari, 2015). Such strategy could be achieved through encapsulating peptides into the core of nanostructures, or chemically conjugating peptides over the surface of nanostructures, thus obtaining peptide-enabled nanostructures with significant gain.

The synergism of peptides and nanostructures could strengthen their favorable characteristics of each technology and overcome natural limitations of individual materials. Many efforts for constructing various peptide-enabled nanostructures for targeted drug delivery have been reported over the past few decades. In this review, we mainly provide an overview of recent advances in the synergism of nanostructures with receptor-targeting peptides that lead to enrichment of biofunctions of nanostructures and reduction of material limitations. It should be noted that due to the limited space, we will outline some representative peptide-enabled targeted delivery systems and related applications in cancer therapies. In order to have a comprehensive view, interested readers are encouraged to consult other related articles and reviews in this issue.

## The Construction of Peptide-Enabled Nanostructures

### Conjugation Strategies

**FIGURE 1:**
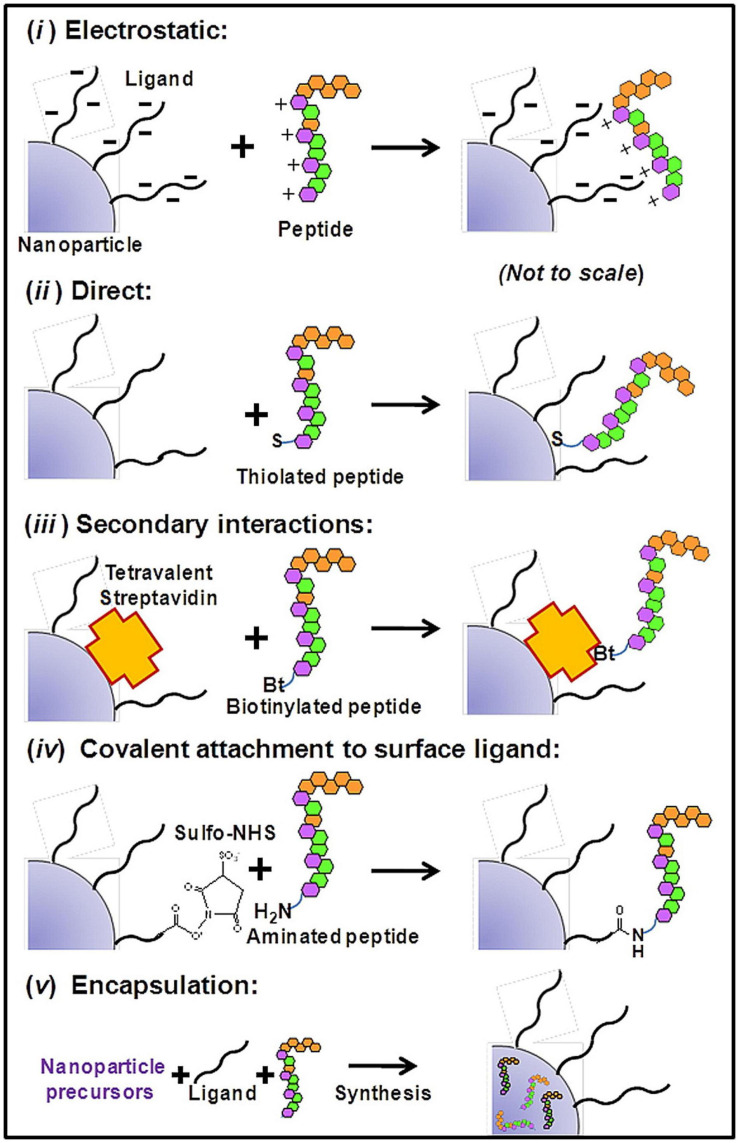
Schematics of generally used bioconjugation attachment generally to NPs: **(i)** Electrostatic interaction. Peptides and NP surface with opposite charges can mediate charge-charge-based attachment; **(ii)** Direct interaction. Some amino acids (e.g., Cysteine) can bind to the surface of AuNPs with high binding affinity *via* Au-thiol dative bonds **(iii)** Secondary interactions. Specific non-covalent interactions such as biotin-streptavidin interactions can mediate direct binding of biotinylated peptides to SA-functionalized NPs **(iv)** Covalent chemical attachment. Classical conjugation chemistry include the conjugation of maleimide with thiols, condensation of carboxyls with amines mediated by NHS/EDC, and various click-chemistry reactions. **(v)** Encapsulation. Such self-assembled encapsulation may be driven by hydrophobic interaction (Sapsford et al., 2013). Copyright 2013 American Chemical Society.

In general, biomolecule-nanoparticle conjugates will either be covalent or non-covalent in nature. The former includes covalent coupling of biomolecules to the surface or surface ligands of nanostructures, while the latter includes non-covalent encapsulation and self-assembled nanostructures driven by non-covalent interaction (Figure 1).

The covalent conjugations of peptides and other biomolecules to the surface of nanoparticles (NPs) are usually driven by the chelation of metal ions or Au-thiol dative bonds. The conjugation to surface ligands of NPs is usually accomplished through functional group coupling reactions. Common examples include the conjugation of maleimide with thiols, and the condensation of carboxyls with amines mediated by *N*-hydroxysuccinimidyl (NHS) along with carbodiimide (EDC). However, when there are multiple reaction groups on the ligands, it will lead to diverse patterns of the connection, which often affects the expected bioactivity. So, many researchers utilized orthogonal click reactions to construct multifunctional NPs. Functional groups that do not naturally exist in endogenous proteins, such as the azido group and alkenyl group, are introduced into the ligands. In this way, conjugation with higher selectivity, more controllable orientation, and fewer by-products can be obtained (Beal and Jones, 2012).

In general, cysteine-containing peptides can be linked to the surface of AuNPs directly. However, such binding may affect the bioactivity of peptides, therefore a sulfhydryl connecting piece is usually added between peptides and NPs. Commonly utilized linker includes cysteine, cystine, mercaptopropionic acid, and glutathione, etc. (Gao et al., 2012). Moreover, since dative bonds are reversible and weaker than typical covalent bonds, monothiol coatings drastically affected by ligand exchange during blood circulation (Kassam et al., 2006). Therefore, peptide coatings with multi-dentate binding sites of NPs have been increasingly explored. Such peptides possess multiple cysteine residues or bidentate dithiol ligands, providing multiple anchoring points to the surface of NPs (Krpetic et al., 2009; Xu et al., 2012). It could be anticipated that such strategy could lead to higher propensity for reattachment and reformation of the initial construct.

In most cases, non-covalent attachment between biomolecules and NPs refers to electrostatic interaction, hydrophobic interaction, hydrogen bond, and π-π stacking forces. For instance, amphiphilic molecules, often lipids, can self-assemble into diverse nanostructures under specific conditions, depending on their natural properties, recognition, and interaction with solvent molecules. The stabilities of non-covalent bindings are dictated by equilibrium dissociation constants so that biomolecule-nanoparticle conjugates are quite sensitive to the concentrations of NPs and biomolecules during preparation and application. These approaches provide advantages such as controllable self-assembly preparation and drug release (Ma et al., 2016). In addition to non-specific physical binding, there are some highly specific and stable non-covalent binding modes, such as the specific interaction between biotin and streptavidin. However, although avidin-biotin coupling has been widely used in the formation of an essentially irreversible and specific linkage between biological macromolecules, this conjugation strategy still has some drawbacks that need to be optimized, such as non-specific interactions for diagnostic assays.

Compared with non-covalent bonding, covalent bonding is more stable. When covalent conjugates are introduced into the complicated biological environments, the targeting ligands covalently coupled to nanoparticles are hard to be destroyed or covered up. However, as for non-covalent conjugates, targeting ligands are more likely to be replaced by various proteins in biological environments. On the other hand, such non-covalent conjugates provide advantages such as easy preparation, usually requiring only stoichiometric mixing of each component. Also, the property of revisable dissociation makes them suitable for designing controlled drug release systems.

### Interaction Between Biomolecules and Interface of Nanostructures

The *in vivo* performance of NPs is closely related to many parameters. Understanding the interaction between nanostructures and biological systems is of fundamental significance. The chemical composition, distribution characteristics, and regulation mechanism on nano-biological interfaces provide the basis for the successful construction of expected biological nanostructures. In general, in addition to natural covalent or non-covalent interaction between biomolecules and nanostructures mentioned earlier, the size and shape of nanostructures, along with the ligand length and ligand density, contribute a lot to their interaction.

For example, many researches showed that the cover ratio of surface molecules will increase with the decrease of the size of NPs (Katari et al., 1994; Chen and Kimura, 1999). These observations may be due to the fact that when the particle size decreases, the radius of the particle begins to contribute to the minimum space required by a surface molecule (Terrill et al., 1995; Love et al., 2005). In addition, the shape of NPs also regulates the adsorption of surface molecules. Manna et al. (2003) demonstrated that organic surfactants would preferentially coat specific faces of a crystal and decreased the energy of these faces. By this effect, they successfully achieved controlled growth of Co nanodiscs and CdSe nanorods (Manna et al., 2003). Nakano et al. (2014) using nucleic acids as model ligands, and demonstrated that the density of ligands on a material surface is not the higher the better. A modest probe density would provide maximum hybridization rate and efficiency, which represents the best binding affinity (Nakano et al., 2014). Another interesting example is the melittin, a positive antimicrobial peptide of 26 amino acids. At low concentrations, the peptide is monomeric and irregularly curled. Once associated with lipid membranes, melittin rearranges by hydrophobic interactions and assumes an α-helical conformation. By investigating the interaction of melittin with phospholipids in liposomes, lipid disks, and micelles, the results showed that melittin tended to adsorb on the highly curved edge of lipid disks (Lundquist et al., 2008).

When there is more than one kind of ligand, the situation becomes more complex. For instance, Kuna et al. (2009) demonstrated that mixed ligand molecules have unique assemble modes on the surface of AuNPs. The ligand mixture self-assembled into large homo-ligand domains, and further self-assembled into stripe-like domains on AuNPs. These stripe-like domains belong to a unique phase separation mode. Many other researches explored the influence factors of the self-assemble structures and phase separation structures of molecules on the surface of NPs, and indicated that the ligand length, ratio of different ligands, compatibility of ligand terminal groups, volume of terminal functional group, and curvature radius of the local surface could influence the phase separation structures (Jackson et al., 2004; Singh et al., 2007, 2011; Tung and Cacciuto, 2013; Yu et al., 2020; Wang et al., 2021). In general, the performance of ligand-decorated NPs is related to many parameters, not only the natural characteristics of NPs and targeting ligands, but also their proportion and connection modes.

## Functional Enhancement of Nanoparticles Enabled by Peptides

### Peptide-Enhanced Biological Stability of Nanoparticles

In order to reach the target site and achieve expected functions, NPs should circulate for enough time in the bloodstream, while avoiding premature removal by the RES and renal clearance. Albanese et al. showed that AuNPs become agglomerated when absorbed serum proteins under physiological conditions, leading to different cellular uptake patterns between single and aggregated nanoparticles (Albanese and Chan, 2011). Besides, the cores of some commonly used NPs are usually heavy metallic and should be capsuled carefully to prevent the leaching of harmful metal ions. Therefore, peptide coatings that could cover metal cores to enhance stability and reduce toxicity are important components of NPs for approaching practical clinical application (Libralato et al., 2017).

The peptide modification onto the surface of NPs has been widely employed to provide biological stabilization to NPs. Common strategies mainly include adjusting the surface charge and hydrophobicity, using biological anti-phagocytic signaling to enhance retention, or promoting the formation of a favorable protein corona such as a pre-formatted albumin corona to prevent the attachment of alternative proteins (Spicer et al., 2018). For example, reduced glutathione has been widely used for the synthesis of AuNPs with good monodispersity and stability (Wu et al., 2014). The functionalization of the peptide of CALNN could endow AuNPs with stability by negatively charged peptide corona to withstand aggregation (Levy et al., 2004). Multi-dentate peptides such as the hexa-histidine motif (His_6_) can form stable metal coordinates with AuNPs and quantum dots (QDs), providing a stable coverage with excellent resistance to desorption (Prasuhn et al., 2010; Aldeek et al., 2013).

### Peptide-Promoted Cell Penetration and Targeting of Nanoparticles

To accumulate into the site of interest, NPs should overcome a series of biological barriers. They need to escape the clearance of the body’s immune system, bypass vascular endothelia, accumulate into the target tissue, and finally recognize and enter into the target cells. Since these biological barriers help the biological system to maintain controlled and ordered material transport, the target tissue and cell penetration of foreign NPs remains one of the major challenges for therapeutic applications (Blanco et al., 2015). In recent years, many cell penetrating peptides (CPPs) with the ability to promote receptor-mediated endocytosis and the following endosomal escape have been widely elaborated (Xie et al., 2020). It is estimated that over 800 of CPPs have been studied (Gautam et al., 2012). The most commonly used CPPs are highly cationic and bind to negatively charged cell membrane through the electrostatic interaction, and then mediate internalization *via* either passive diffusion or endocytic pathway. Typical examples of CPPs include the TAT (Schwarze et al., 1999), R_8_ (Yang et al., 2018), penetratin (Jiang et al., 2019), and pHLIP peptide (Thevenin et al., 2009).

For instance, the TAT (GRKKRRQRRRPQ) sequence is originally derived from the human immunodeficiency virus and has been demonstrated to deliver drug-loaded nanocarriers including liposomes, polymeric micelles, and metal NPs into cells. Interestingly, the D-enantiomer of TAT and disordered TAT sequence also show the ability to mediate cell penetration (Futaki et al., 2001; Qin et al., 2012), suggesting that the positive charges of CPPs play a vital role in enhancing cell internalization, rather than specific receptor-induced internalization (Futaki et al., 2001; Qin et al., 2012). Importantly, although cationic peptides enhance cell uptake efficiently, endosomal escape may be hindered by the strong electrostatic attraction, which may partly account for the fact that the efficiency of cargos to deliver to the cytosol remains relatively low (Verdurmen et al., 2017). To enhance endosomal escape efficiency, Morshed et al. (2016) attached TAT peptide to AuNPs *via* an acid-labile hydrazine bond that would cleave spontaneously after reaching acidic endosomal environments, which greatly promoting the endosome escape of AuNPs. In general, for designing CPPs-functionalized NPs, the balance of cell penetration and endosomal escape ability should be considered carefully.

### Peptide-Promoted Tumor Penetration and Targeting of Nanoparticles

Numerous peptides named tumor homing peptides have been widely identified. They have the potential to penetrate tumor tissues, resulting in an extensive distribution across the tumor mass. For example, Teesalu et al. reported a class of peptides (named as CendR) that share an (R/K)XX(R/K) motif in the C-terminus and specifically target the neuropilin-1 receptor, a cell membrane receptor that regulates vascular permeability and angiogenesis, as well as the development of the nervous system (Teesalu et al., 2009). Interestingly, this motif must be exposed at the C-terminus of the peptides to remain the biological activity. The CendR sequence can be embedded in a long peptide sequence and activated by an appropriate protease cleavage.

Up to date, the RGD sequence and its cyclic derivative (cRGD) might be the most widely used tumor homing sequences, which direct to integrin α_v_β_3_, a signaling protein that is overexpressed in many types of endothelial tumors (Desgrosellier and Cheresh, 2010). To show the synergy between the two peptides, Sugahara et al. (2009) constructed a cyclic fusion sequence of c(CRGDK/RGPD/EC) that triggers tissue penetration of drugs through three processes: The RGD motif targets integrin αvβ3 to accumulate at tumor endothelium cells, then exposes the cryptic CendR motif, RGDK/R, by proteolytic cleavage, and finally induces tumor penetration following interaction with neuropilin-1. In recent years, RGD modified NPs have been exploited rapidly. Xie et al. developed cRGD-decorated semiconducting polymer nanoparticles for photoacoustic imaging (Xie et al., 2017). The results showed that with the targeting capability of cRGD, NPs could effectively delineate the tumor sites in living mice and increased the photoacoustic intensity. Fan et al. also demonstrated that RGD modification could enhance the specific targeting ability of self-assembled fluorescent nanoparticles and improve their anti-tumor activity (Fan et al., 2018).

In addition to tumor homing peptides, a variety of peptides that has high binding affinities to target receptors overexpressed on specific cells have been widely used to realize cell targeting. The GE11 peptide (YHWYGYTPQNVI) is a typical example, which is firstly identified by Li et al. (2005) through the screening of phage display libraries. The GE11 peptide has been demonstrated to promote tumor penetration and selectively bind to the epidermal growth factor receptor (EGFR), a typical overexpressed receptor in epithelial origin tumors. A series of subsequent researches confirmed that GE11 could act as an excellent allosteric EGFR ligand without mitogen activity. GE11 peptide-mediated drug delivery systems including liposomes, polymeric micelle, and viruses have been developed for disease diagnosis and drug delivery. A summary of GE11 peptide-enabled targeted delivery systems has been elaborately discussed in the review of Genta et al. (2018).

In most cases, selected ligands will promote the specific accumulation at targeting sites. For example, encouraged by our previous work that peptide E5 (GGRSFFLLRRIQGCRFRNTVDD) was first identified as an antagonist to chemokine receptor CXCR4 with effective anti-metastasis activity (Li et al., 2014). In a subsequent study, Duan et al. (2016) demonstrated that E5 can also bind to human serum albumin (HSA) with a high affinity and presented enhanced interaction between HSA and CXCR4-overexpressed tumor cells mediated by E5. Compared with free HSA, the *K*_D_ value of E5-HSA nanocomplex to CXCR4-positive cells improved by approximately an order of magnitude, and showed improved cell migration inhibitory effect. Their results indicate that a single peptide sequence can be multi-functional. On one hand, E5 can serve as a targeting ligand to endow nanostructures specific binding affinity to CXCR4. Indeed, Zu et al. (2020) subsequently demonstrated that E5 modified QDs also showed much higher binding affinity to Hela cells compared with free E5 (The equilibrium dissociation, *K*_D_: 15.5 μM and 125 nM, respectively). On the other hand, HSA is the most abundant protein in the plasma, and their binding will not affect the normal function of E5. These properties indicate the formation of a favorable protein corona for E5-decorated NPs, preventing the attachment of other alternative proteins. That may partly decrease the unexpected influence of the rebellious protein corona and prolonging the circulation time. Moreover, since E5 is originally a therapeutic peptide with anti-metastasis activity, their synergism provides an excellent platform for affinity-controlled drug release systems. On the whole, this study has shown that compared with passive targeting NPs, the peptide ligand-directed NPs present improved therapeutic performances in various degrees. However, it is worth noting that the development of ligand directed NPs remains in its infancy. At present, there was no ligand-directed active targeting nanomedicine has been approved for clinical use.

### Peptide-Enabled Controlled Drug Release of Nanoparticles

Peptide-drug conjugates (PDCs) are an emerging strategy for delivering payloads to target tissues while decreasing unexpected effects to healthy tissues. Drugs are covalently attached to specific peptides *via* cleavable linkers to make prodrugs. This could temporarily mask or limit the drugs’ bioactivity and minimize premature drug liberation. The diversity of peptide sequence can not only enable the facile preparation of various kinds of PDCs with unique specificity, but also regulate the hydrophobicity and ionization of the whole conjugates, which contribute greatly to the bioavailability. At present, there are two PDC drugs (Lutathera and Melflufen) that have been approved by Food and Drug Administration (FDA) and many candidates are being evaluated in various stages of clinical development (Cooper et al., 2021). In this field, advanced design is so-called one-component nanomedicine, which means amphiphilic peptide-drug conjugates self-assembling to form their own nanostructure as drug delivery vehicles (Ma et al., 2016). Active drugs can release in control over time or initiate by some specific stimulus after introducing into biological environments.

For example, Yang et al. (2019) fabricated a self-assembling active targeting anticancer hydrogel by conjugating both chlorambucil and tyroservatide to a self-assembling peptide sequence. They demonstrated that a heating-cooling process would easily urge the compound monomer to self-assemble into a nanofiber structured hydrogel. The hydrogel could concurrently deliver the two drugs with controlled release and prolonged plasma circulation half-life, leading to enhanced cell uptake and antitumor activity.

Peptides can also be utilized for engineering affinity-controlled release systems, which could achieve controlled diffusion of drugs through the different binding affinities between molecules (Pakulska et al., 2016). In general, elaborated drug-binding ligands, such as peptides, oligonucleotides, or proteins, are immobilized onto a polymer matrix. Then therapeutic drugs can bind to these ligands through preferred non-covalent interactions. An equilibrium is established between free drugs and ligand-bound drugs. By adjusting the concentration of the ligand and the binding strength of the ligand with the drug, the rate of drug release can be regulated to meet different requirements. In this field, a series of potential affinity peptides have been identified by *in vitro* selection and directed evolution. Now a database of affinity peptides (MimoDB) (Huang et al., 2012) has been established, which may contribute to affinity-controlled release applications.

## Peptide-Enabled Nanostructures of Targeted Delivery Systems

### Peptide-Mediated Liposomal Delivery Systems

Liposomes are closed spherical vesicles with a lipid bilayer structure, which is similar to the cell membrane. This unique structure allows effective encapsulation of both hydrophilic and hydrophobic molecules. Such encapsulation improves the stability of loaded drugs and reduces their systemic toxicity. Liposomes provide substantial advantages over many other nanocarriers, including consistent size, efficient drug loading, excellent steric stabilization, and biocompatibility (Noble et al., 2014). In the past few decades, liposome-based nanomedicines have been considerably explored. Several liposomal drugs have achieved efficacious clinical outcomes (Crommelin et al., 2020). For instance, the liposomal doxorubicin (Doxil®) is the first FDA-approved liposomal therapy for cancer treatment, providing improved survival rate and life quality for patients over free doxorubicin (Barenholz, 2012).

The liposomal delivery system can be facilitated by targeting peptides to mediate specific drug delivery, leading to increased drug penetrability and specific accumulation at the site of interest. Such sequence can be easily functionalized to the surface of liposomes *via* reactive lipid head groups, to obtain peptide-enabled targeted drug-loaded liposomes. After extravasating into the tumor interstitial space *via* the EPR effect, liposomes can be internalized into specific target cells by ligand-receptor recognition. These peptide-functionalized liposomes combine both passive and active delivery mechanisms, which could exhibit better drug delivery efficiency than drug-containing liposomes without peptide modification (Wilhelm et al., 2016). Until now, a variety of engineered liposomes by the introduction of targeting peptides have been successfully designed to deliver cargoes to specific organs, tissues, and tumor cells through multiple targeting (Jiang et al., 2012; Roy et al., 2017; Huang et al., 2018; Lu et al., 2019).

**FIGURE 2:**
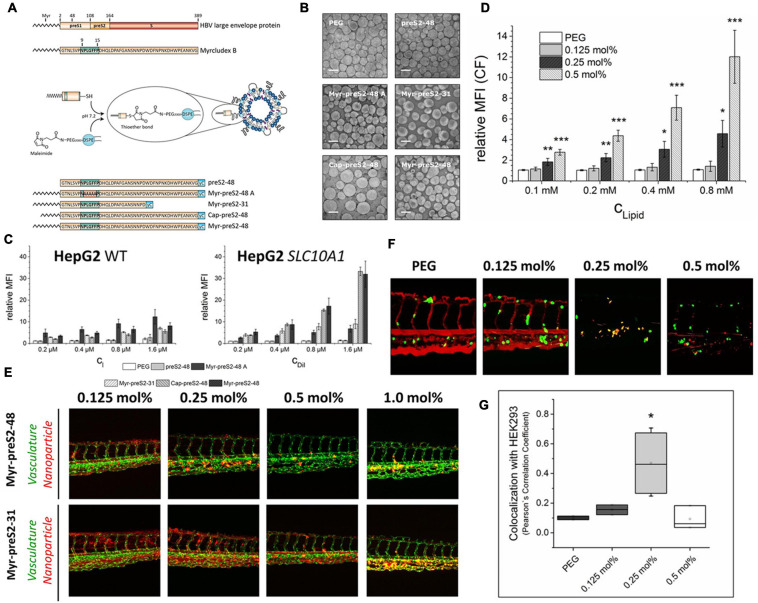
Identifying different peptide ligands for a better NTCP-specific targeting. **(A)** Schematic representation of HBV large envelope protein, Myrcludex B and its five derivatives. Peptides were conjugated to the end of PEG chain integrated in the liposomes through the connection of sulfhydryl group with maleimide group. **(B)** The morphological characters of different Myrcludex B-derived lipopeptide conjugated liposomes. **(C)** The uptake rate of different peptide conjugated liposomes into HepG2 wild type cells and SLC10A1 overexpressing HepG2 cells was identified by flow cytometry analysis. **(D)** The uptake of liposomes modified with different amounts of Myr-preS2-31 identified by flow cytometry analysis. Significance (*p < 0.05, **p < 0.01, and ***p < 0.001) was calculated relative to PEG, respectively. **(E)** The performance of Myr-preS2-48- and Myr-preS2-31-modified liposomes was tested in zebrafish embryos expressing green fluorescent protein in their vasculature endothelial cells (green signal). DiI (red signal) was used to label the membrane of liposomes. **(F)** Myr-preS2-31-modified liposomes were tested in wild type zebrafish embryos xenotransplanted with human, GFP expressing HEK293 cells (green signal), expressing SLC10A1. DiI (red signal) was used to label the membrane of liposomes. Yellow signals demonstrate the colocalization of liposomes with HEK293-GFP cells. **(G)** Pearson’s Correlation Coefficient (PCC) for quantitative analysis of liposomes binding to HEK293-GFP cells (Witzigmann et al., 2019). Significance (*p < 0.05) was calculated relative to PEG. Open Access.

For instance, in order to design a hepatotropic liposome, Witzigmann et al. (2019) selected and optimized a derivative peptide of Myrcludex B that derived from the hepatitis B virus large envelope protein. Myrcludex B-derived peptides could specifically target the sodium-taurocholate cotransporting polypeptide (NTCP/SLC10A1) that overexpressed on the hepatocyte sinusoidal membrane (Yan et al., 2012; Witzigmann et al., 2019). On this basis, they further designed five Myrcludex B-derived peptides with different sequences and acyl chain modifications, and modified these peptides onto the surface of liposomes (Figure 2A). The shorter fatty acid chain (Cap-preS2-48, C10) seems to lead to more differentiated and unstable liposomes (Figure 2B) than the longer fatty acid chain (Myr-preS2-48, C14). This phenomenon may be attributed to the shorter acyl chain interferes with the liposome stability by backward bending insertion into liposomal membranes. Some earlier researches also have shown that shorter lipid anchors were less stable in liposomal membranes, promoting faster dissociation and re-association with neighboring lipids (Webb et al., 1998; Sauer et al., 2006). As shown in Figure 2C, the cellular uptake of Myr-preS2-48A modified-liposomes is not related to the level of NTCP expression, suggesting that NPLGFFP plays an important role in NTCP-specific targeting. In addition, the longer fatty acid chain modification onto the surface of liposomes at the end of the peptide represents the higher binding affinity to NTCP-overexpressing HepG2 cells. The peptide of preS2-48 without fatty acid modification did not possess a considerable binding affinity to NTCP receptors.

Subsequently, the peptide ligand density of liposomes can be regulated by the added amounts of coupled maleimide group of PEG_2000_-DSPE chain. The results showed that the cellular uptake of peptide modified liposomes is ligand concentration-dependent, and at least 0.25 mol% of ligand concentration (Myr-preS2-31) is needed for efficient active targeting (Figure 2D). When Myr-preS2-31- and Myr-preS2-48-modified liposomes are introduced into the transgenic zebrafish embryos at identical ligand densities, liposomes modified with shorter peptides (Myr-preS2-31) show better systemic circulation compared with longer ligand modified liposomes (Figure 2E), which may because the longer sequence indicates higher immune clearance. Similarly, the decreased systemic circulation time and higher clearance rate also counteract the advantage of higher ligand densities (0.5 mol%) (Figure 2F). Under the comprehensive balance between the target capability and biocompatibility, a 0.25 mol% of Myr-preS2-31 ligand modification would exhibit the best performance (Figure 2G).

On the whole, various elements can influence the interaction between nanomaterials and bio-interface. That may partly account for the fact that all clinically approved liposome-based nanomedicines are non-ligand directed, which totally relies on passive targeting to achieve tumor accumulation. Although many positive results have been reported in animal models, few positive effects in patients were published for such ligand-targeted liposomes (Crommelin et al., 2020). It is laborious but important to carefully evaluate these effects and search for the best balance.

Similarly, Zou et al. (2018) developed a vemurafenib-loaded liposome for the precise inhibition of subcutaneous melanoma via the skin. The TD peptide (ACSSSPSKHCG) endowed liposomes with the ability to target subcutaneous melanoma cells harboring BRAF mutation. It can temporarily open the paracellular pathway and promote the drug to penetrate the skin (Chen et al., 2006). Compared with oral administration or intravenous injection, transdermal delivery performed a better biological effect in enhancing antitumor efficacy and reducing damage to normal organs.

Zhao et al. (2020) described a double-modified doxorubicin-encapsulating liposome (AAN-FnBPA5-Dox). The AAN motif was covalently bind to the N-terminal of the FnBPA5 peptide. Then, such tandem peptide was covalently linked to the doxorubicin-encapsulated liposomes *via* amide bonds. The FnBPA5 peptide has a high binding affinity to relaxed Fn and collagen I in the extracellular environment and α-SMA-expressing cancer-associated fibroblasts, reducing the density of collagen fibers around the tumor tissue, and promoting the cell penetration of doxorubicin. And the AAN motif was attached to extend the circulation time by hindering parts of the bioactivity of FnBPA5 sequence and reducing the accumulation in off-target Fn-excreting organs. The results indicated that AAN-FnBPA5-Dox remained stable during internal circulation until the AAN motif was recognized and cleaved by legumain that overexpressed in tumor-associated macrophages (Song et al., 2016). In general, such modification would partly hinder the natural bioactivity of the cryptic sequence until the cover motif was cleaved. An earlier study claimed that the addition of the AAN moiety to TAT sequences would lead to a decrease transport efficacy by 72.65% until legumain catalysis recovered the penetrating capacity (Liu et al., 2014). In this way, they successfully formed an ecological therapy, which could extend the circulation time, regulate the tumor microenvironment, thus improve the therapeutic response.

### Peptide-Mediated Polymeric Micelle Delivery Systems

Polymer micelles usually refer to self-assembled spherical colloidal nanoparticles formed by amphiphilic block copolymers. They usually possess a core-shell structure in an analogous fashion to liposomes in which poorly soluble cargos can be encapsulated. In terms of drug delivery, micellar-based systems are suitable for carrying hydrophobic molecules. The advantages of these structures include a simple preparation process, efficient drug loading, well biocompatibility, and controlled release kinetics (Long et al., 2015). The physical properties and biological activity of micelles can be regulated by the functionalization of flexible pendant groups. This makes it possible for synthesizing stimuli-responsive polymeric micelles that could be controlled to release cargos in response to various surroundings (Aura-Ileana et al., 2012). Thus, polymeric micelles are finding increasing utilization across many biomedical disciplines. However, it should be noted that a practical issue is that polymeric micelles tend to dissociate and release cargos when they are diluted and interact with biomolecules in the blood, often leading to premature cargo release (Bae and Yin, 2008).

**FIGURE 3:**
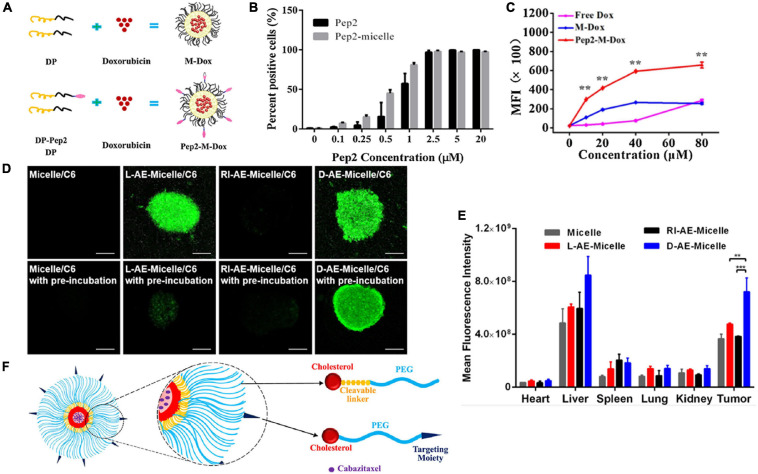
Peptide-mediated polymeric micelles for therapeutic applications. **(A)** Schematics of the preparation of pep2 modified DSPE-PEG_2000_ micelles. **(B)** The binding affinities of micelles with or without pep2 modification to HepG2 tumor cells, quantified by flow cytometry. **(C)** MFIs of HepG2 cells treated by Free doxorubicin, Micelle-doxorubicin, or pep2-Micelle-doxorubicin at equivalent concentrations of doxorubicin (Zheng et al., 2021). Significance (**p < 0.01) was calculated relative to M-Dox. Copyright 2021 Elsevier. **(D)** Transcytosis of modified micelles in BBB/U87 model *in vitro*. Penetration of different micelles in the BBTB/U87 tumor spheroid coculture model with or without preincubation were examined by a confocal microscope. L-AE: _L_-(FALGEA); D-AE: _D_-(FALGEA); RI-AE: _D_-(AEGLAF). **(E)** The fluorescence intensity of different micelles in tumor and organs were identified by semiquantitative ROI analysis (Mao et al., 2017). Copyright 2017 American Chemical Society. ** and *** indicate *p* < 0.01 and *p* < 0.001, respectively. **(F)** Schematic illustration of the MMP-2-responsive polymeric micelle (Barve et al., 2020). Copyright 2020 Elsevier.

For instance, Zheng et al. (2021) developed a peptide-directed micelle delivery system targeting tumor-associated antigen of CD36. Co-assembled DSPE-PEG_2000_ micelles were modified with targeting peptide pep2 (RRGTIAFDNWVDTGTRVYD) and thus obtained appreciably enhanced specificity and sensitivity toward CD36-positive tumor cells. Compared with free pep2, the binding affinity of pep2-modified micelles to the CD36 receptor was enhanced by ∼30% (Figures 3A,B). Relatively, as shown in Figure 3C, compared with unmodified micelles (M-Dox), the intracellular delivery amount of doxorubicin was enhanced ∼3-fold with the modification of pep2 (Pep2-M-Dox).

Another similar study utilized a D-peptide ligand for better proteolytic stability and demonstrated that the modification of micelles with D-peptide ligand could facilitate micelles to pass through the simulated blood-brain barrier and thus achieve deeper drug penetration (Mao et al., 2017). As shown in Figures 3D,E, compared with micelles without peptide modification, FALGEA modification showed higher recognition ability to EGFR-positive tumor cells, while the reverse sequence of FALGEA did not have such specificity. Moreover, compared with L-type FALGEA, the D-type FALGEA-modified micelles exhibited enhanced stability and targeting ability both *in vitro* and *in vivo*.

In addition to surface modification to promote specific tumor recognition and accumulation, peptides can also be encapsulated within micelles as a switch for drug directed release. Barve et al. developed a biodegradable, enzyme-responsive micelle with a block copolymer consisted of PEG, a matrix metalloproteinase-2 (MMP-2)-targeting peptide (PLGVRK), and cholesterol (Figure 3F; Barve et al., 2020). When the system was introduced into MMP-2 overexpressed tumor microenvironment of prostate cancer, the linker between cholesterol and PEG chain would be cleaved, which induces micelle dissociation and subsequent drug release of cabazitaxel. With this design, a controlled drug delivery system responding to the tumor microenvironment was constructed successfully.

Another interesting research showed the potential of antigen peptide mediated polymeric micelles as cancer nano-vaccines to activate the body’s active anti-tumor immunity (Liu et al., 2017). Liu et al. designed a type of micelle vaccine incorporating both two kinds of antigen peptides (E7 and OVA) and monophosphoryl lipid A (MPLA) as an immune adjuvant. The E7 (GQAEPDRAHYNIVTFCCKCD) and OVA peptide (SGLEQLESIINFEKL) were selected as tumor-associated antigens to activate specific tumor immune response (Liu et al., 2017). The results demonstrated that PEG-PE micelles could assist non-α-helical structures of E7 and OVA refolding into α-helix structures and enhance their cytosolic delivery, which could exhibit an impressive therapeutic antitumor effect (Fang et al., 2016). Their efforts provide a new design for cancer treatment, which is expected to significantly improve the application and benefit rate of cancer patients.

### Peptide-Guided Exosomal Delivery Systems

Exosomes are a class of nanosized extracellular vehicles (EVs) secreted by living cells, showing great potentials in advanced drug delivery and disease treatment. Fundamentally, EVs are a universal form of signal transduction between cells. They circulate in the blood and transport various bioactive molecules to regulate cell function and behaviors in distant target cells (Kalluri and LeBleu, 2020). Therefore, compared with other artificial NPs, exosomes have a natural ability to deliver therapeutic molecules with advantages including minimal immune clearance and well biological barrier penetration (Barile and Vassalli, 2017). For example, Haney et al. (2015) exploited the ability of exosomes to cross the blood-brain barrier and developed a non-invasive approach to deliver catalase to the brain. They utilized intranasal administered exosomes loaded with catalase to cross the blood-brain barrier, and subsequently obtained promoted neuroprotective effects (Haney et al., 2015). So far, several clinical trials have been carried out to test the safety of exosome-based nanomedicine and some of them have shown satisfying outcomes in dendritic cell-derived exosomes (Pitt et al., 2016).

In recent years, peptide ligand enrichment on engineered exosomes has been investigated to target specific cell types. Compared with other artificial NPs, exosomes have their unique ligand modification strategy. The modification may be achieved by engineering the parent cells, leading to the natural exhibition of ligands on the surface of exosomes. For peptide ligands, their coding sequences can be inserted into a lentiviral expression plasmid and transfect into producer cells, and fused with an EV transmembrane protein, such as Lamp2b or PDGFR TM domain. Then the peptide will naturally display on the outer surface of exosomes produced by these cells (Dang and Zeng, 2016).

**FIGURE 4:**
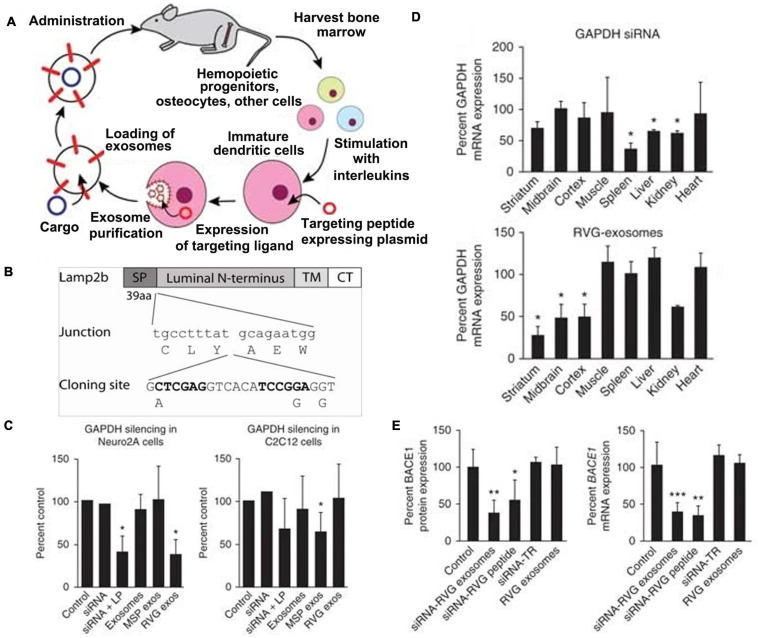
Delivery of siRNA to the mice brain by peptide-mediated exosomes. **(A)** Schematic representation of the preparation of self-exosomes for gene delivery. **(B)** The modified Lamp2b protein. SP, signal peptide; TM, transmembrane domain; CT, C terminus. **(C)** qPCR of GAPDH in neuronal cells (Neuro2A) and murine muscle cells (C2C12) of medium, naked siRNA, siRNA delivered with Lipofectamine 2000, unmodified exosomes, MSP exosomes, and RVG exosomes. Significance (*p < 0.05) was calculated relative to Control group, respectively. **(D)** The GAPDH qPCR in different organs of mice treated by naked GAPDH siRNA or encapsulated siRNA in RVG exosomes, normalized to untreated controls (100%). Significance (*p < 0.05) was calculated relative to untreated controls, respectively. **(E)** The BACE1 qPCR of mice brain cortical sections of five groups of free RVG exosomes, RVG exosomes encapsulated with BACE1 siRNAs, transfection reagent with BACE1 siRNA, and RVG-9R peptide with BACE1 siRNA, and compared with untreated control. All qPCR was normalized to 18S RNA levels. Significance (*p < 0.05, **p < 0.01, and ***p < 0.001) was calculated relative to Control group, respectively (Alvarez-Erviti et al., 2011). Copyright 2011 Springer Nature.

It has been generally proposed that exosomes are suitable for delivering nucleic acids and exchange genetic information between cells, containing both mRNA and microRNA (Valadi et al., 2007). Many researches show that exosomal mRNAs are functional and can regulate the phenotype of target cells (Valadi et al., 2007; Mittelbrunn et al., 2011; Kojima et al., 2018). Thus, engineered exosomes become ideal carriers for target genetic therapy. For example, Lydia et al. (Alvarez-Erviti et al., 2011) explored an endogenous brain-targeting exosome for siRNA delivery (Figure 4A). They cloned the sequence that encodes an acetylcholine receptor targeting peptide (YTIWMPENPRPGTPCDIFTNSRGKRASNG, RVG) into the N terminus of murine Lamp2b protein on the exosomal membranes (Figure 4B), and then introduced the engineered plasmid into dendritic cells to express targeting exosomes. The results showed that these engineered exosomes possessed similar gene knockdown efficiency compared to transfection reagents with significant specificity (Figure 4C). As for the RVG peptide-modified exosomes, they exhibited decreased off-target effects, and great therapeutic potential by the strong knockdown efficacy in both mRNA and protein level (Figures 4D,E).

However, it should be noted that this peptide fusion is difficult to predict and control. Because of the fusion of targeting peptides into membrane proteins to form large compounds, poor expression, adverse folding, and incorrect display of these fusion proteins remain of potential concern. Furthermore, compared with other simpler NPs, exosomes are difficult to prepare, usually isolated from exosome-secreting cell lines by ultracentrifugation, which limits their large-scale application. In addition, the producer cell type, physiological state, and manufacturing protocols will vary the composition and properties of exosomes, making it difficult to characterize exosomes pharmaceutically (Barile and Vassalli, 2017). These characteristics remain the major obstacles in translating naturally secreted exosomes into clinical practice.

### Peptide-Modified Gold Nanoparticles (AuNPs)

Gold nanostructures show outstanding potential with multi-functionality, such as antimicrobial, anticancer, drug delivery, sensing, and imaging. They are relatively inert and stable in biological environments, offering attractive tools for biomedical diagnostic and therapy (Dreaden et al., 2012; Ramalingam, 2019). Particularly, AuNPs exhibit unique localized surface plasmon resonance-related optical phenomena, which show strong light emission excited by concerted electron oscillation. The unique optical and electronic properties of AuNPs make them valuable tools for *in vitro* diagnostics, including near-infrared fluorescence, enhanced magnetic resonance imaging, surface-enhanced Raman scattering, as well as photothermal and photoacoustic imaging (Boisselier and Astruc, 2009). Wu et al. (2019) summarized a brief review of recent advances of AuNPs in bioimaging, and herein, we mainly focus on the therapeutic applications of AuNPs.

Gold nanoparticles possess a unique photothermal effect. They can absorb the light near the NIR region and convert it to heat effectively, causing the death of malignant tumors with the advantage of minimal invasiveness (Beik et al., 2019). Noteworthy, endogenous biomolecules appear the minimal absorb in the NIR diagnostic windows (650∼900 nm, and 1000∼1350 nm) (Kobayashi et al., 2010). This allows photothermal therapy based on AuNPs to penetrate the body more deeply. Indeed, photothermal therapy is a central application of AuNPs and has been employed widely to destroy tumor cells. It is noteworthy that such localized destruction of pathological parts strongly relies on specific and adequate accumulation at the target site. Therefore, an effective targeting ligand such as peptide appears more of significance. Some recent examples referring to peptide-modified AuNPs in photothermal therapy are listed in the Table 1, some of them also loaded with other drugs for combination therapy.

**TABLE 1:** A brief summary of recent AuNPs in therapeutic applications.

**Peptide**	**Target**	**Loading**	**Delivery to**	**References**
KTLLPTPYC	Plectin-1	Gemcitabine	Tumor cell	Pal et al., 2017
TAT	–	Cas9-sgRNA	Tumor cell	Wang et al., 2018
GE11	EGFR	Pc 4	Tumor cell	Meyers et al., 2015
RGD	Integrin αvβ3	–	Tumor blood vessels	Ali et al., 2017
RGD and CGGGPKKKRKVGG	Integrin αvβ3	–	The nucleus of tumor cell	Aioub and El-Sayed, 2016
CRQAGFSL	Urokinase-type plasminogen activator receptor	5-Aminolevulinic acid	pancreatic tumor cell	Li et al., 2017
CPNFDWDPNNSNAGF APDLQHDPFFGLP	Squamous Cell Carcinoma Antigen 1	–	Hepatocellular Carcinoma Cells	Jha et al., 2017
TSFAEYWALLSP	MDM2 and MDMX	–	Tumor cells bearing wild-type p53	He et al., 2020

Peptide-enabled AuNPs have also been used to direct payloads to their targets. Pal et al. reported a kind of AuNPs for plectin-1 targeted gemcitabine delivery in pancreatic cancer (Pal et al., 2017). Plectin-1 is aberrantly overexpressed on the surface of pancreatic ductal adenocarcinoma while showing cytoplasmic expression in healthy cells. They used a multifunctional peptide sequence (KTLLPTPYC) as the targeting ligand, as well as the reducing agent. Such sequence was derived from a plectin-1 targeting sequence (KTLLPTP). A tyrosine residue is attached at the C-terminal for reducing property while the cysteine residue is attached for coupling with AuNPs. This modified peptide plays the role of glutathione in traditional synthesis, to fabricate AuNPs via an *in situ* reduction, simplifying the synthesis process effectively. These AuNPs were further functionalized with gemcitabine through electrostatic interaction of the pyrimidine ring or reversible Au-N bond, which accounted for an initial burst drug release followed by a sustained release. In subsequent *in vivo* experiments, the sequence KTLLPTP promoted the spacer selectively accumulation in tumor tissues and thus leading to higher specific cytotoxicity to pancreatic ductal adenocarcinoma cells than chemotherapeutic drugs alone.

## Challenges in Peptide-Promoted Delivery Systems for Tumor Therapies

At present, almost all clinically approved drug delivery systems are non-ligand directed, which totally relies on passive targeting to achieve tumor accumulation. Although many positive results have been reported in animal models, few positive effects on efficacy in patients were published for ligand-targeted NPs (Crommelin et al., 2020). Key challenges of clinical transformation include inadequate standard methods for clinical production and comprehensive characterization, an overall understanding of NPs function and behavior in the body, and the difficulties in the recapitulation of natural tumors in laboratory models (Belfiore et al., 2018). Some of them are waiting for technique development and some rely on foundational research. Herein, we focus on the potential of peptides in overcome such difficulties and highlight that a proper peptide coating may influence and regulate the formation and composition of a serum protein corona on the surface of NPs, which may contribute to a better performance *in vivo*.

The biophysical properties of NPs-based delivery systems after intravenous injection are an important consideration, including ligand stability, ligand function, circulation time, and clearance properties. After the introduction of NPs into the biological environment, the nanoparticle surface will rapidly interact with a mixture of biomolecules especially proteins to form a layer of the protein corona, which is dynamic and highly dependent on the biological environment (Monopoli et al., 2012). High abundant proteins form an initial corona and are gradually removed by tightly bound proteins to form thermodynamically favored hard corona. The performance of the protein corona is difficult to predict and often leads to unexpected influences of the fate of NPs *in vivo* with undesired activity or function (Corbo et al., 2017). It has been generally proposed that the presence of a protein corona around the surface of peptide-enabled targeted delivery systems may inhibit the binding affinity of targeting ligand with its receptor, reducing the targeting ability of the NPs, accounting for unanticipated biodistribution, pharmacokinetics, and efficacy *in vivo* (Walkey and Chan, 2012; Salvati et al., 2013).

In this field, a proper coating may modulate and control the formation and property of the protein corona. In general, opsonins such as immunoglobulin G, complement factor and fibrinogen, can be adsorbed on the surface of NPs and trigger macrophage recognition and phagocytosis elimination (Owens and Peppas, 2006; Gao and He, 2014). If this process is inhibited, the recognition and clearance of NPs by the immune system can be reduced. On the contrary, when dysopsonins such as serum albumins and lipoproteins are enriched on the surface of NPs, the recognition is blocked and the circulation time can be increased (Peng et al., 2013). In the past few years, stealth polymers such as polyethylene glycol (PEG) have been widely used for limiting opsonins adsorption and decreasing NPs clearance. Such stealth polymers can form a protective layer around the NPs and provide a steric barrier to adsorption (Moore et al., 2015). Some of them can also regulate the surface charge and hydrophobicity for better biocompatibility. In addition, peptides with high binding affinity to dysopsonins may promote the formation of a beneficial protein corona, which could decrease the rapid clearance of NPs. Chen et al. (2009) demonstrated an ultra-low fouling peptides with alternating positive (K) and negative charge (E) residues. Such mixed charged sequence possesses strong hydration ability and leads to a hydrated layer on the surface of NPs, preventing non-specific protein adsorption. However, there is a contradiction between the stealth performance and targeting performance. Many researches show that a stealth coating is associated with reduced specific cellular uptake (Yu et al., 2012). On the whole, it is important to consider the delicate balance between biocompatibility and targeting capability that determines NPs fate when designing a modification coating. More peptide sequences with expected characteristics are waiting for design, selecting and identification.

## Conclusion

Nanotechnologies have developed rapidly, and targeting peptides have also found increasing favor in this context. The synergism combines favorable characteristics of nano-sized structures with bioactive peptides, which could enrich the biofunction, and overcome the natural limitations of individual materials. Over the past few decades, nanoparticles conjugated with peptides have emerged as powerful tools for biomedical applications. Numerous peptide-functionalized nanomaterials have been developed for biomedicine applications, including drug delivery, biological imaging, liquid biopsy, and targeted therapies. Nanotechnology has already revolutionized the mode that we discover and administer new biomedicine. However, obstacles such as inadequate knowledge about nanostructure interacting with biological interfaces still remain profound. Cautious design and further exploration to improve the safety and efficacy of these nano-formulations would be a necessary and long-term project.

## Author Contributions

ML: conceptualization, data curation, and writing-original draft. XF: validation, writing-review and editing, and funding acquisition. YY and CW: conceptualization, resources, writing-review and editing, supervision, and funding acquisition. All authors contributed to the manuscript and approved the submitted version.

## Conflict of Interest

The authors declare that the research was conducted in the absence of any commercial or financial relationships that could be construed as a potential conflict of interest.
